# Digital finance and Chinese corporate labor investment efficiency: The perspective of financing constraints and human capital structure

**DOI:** 10.3389/fpsyg.2022.962806

**Published:** 2022-08-10

**Authors:** Jing Yang, Yalin Jiang, Hongan Chen, Shengdao Gan

**Affiliations:** ^1^School of Business, East China University of Science and Technology, Shanghai, China; ^2^School of Social Audit, Nanjing Audit University, Nanjing, China; ^3^School of Business, Sichuan University, Chengdu, China

**Keywords:** digital finance, labor investment efficiency, financing constraints, human capital structure, instrumental variable

## Abstract

As the aging population problem intensifies, many emerging economies are caught in labor shortage and rising labor costs, thus improving the corporate labor investment efficiency (LIE) is crucial for these countries. In this context, we take China as an example to explore the influence of the current booming digital finance (DF) on corporate LIE. This paper, which enriches the existing literature, is one of the few studies that explores the link between macroeconomic policies and firms’ LIE. Our research adopts the baseline methodology of ordinary least squares (OLS) regression, and the data comprise 23,503 observations for Chinese A-share listed businesses from 2011 to 2020. In addition, we use fixed effects regression, instrumental variables method and substitution of independent variables to deal with endogeneity and test the robustness. The outcomes suggest that DF may significantly increase corporate LIE. Further results from the path mechanism study suggest that DF could alleviate financing constraints and optimize human capital structure, both of which have a favorable effect on the LIE. Last but not least, the heterogeneity results imply that DF can more effectively encourage LIE of firms in economically underdeveloped regions and of private nature. The study recommends that emerging economies should pay attention to strengthening regulation to avoid financial risks while vigorously promoting DF. In addition, enhancing the level of human capital and optimizing human capital allocation are also essential.

## Introduction

Labor, as one of the most crucial factors in the production and operation of firms (Hamermesh, 1993), accounts for around two-thirds of the global economic added value (Jung et al., 2019b). Moreover, due to strong externality, labor can break through the shackles of the law of diminishing marginal returns and thus contribute to long-term healthy economic growth (Lucas Jr., 1988), so effective labor investment has the role of enhancing corporate production capacity, improving revenue output efficiency, and thus boosting the profitability of firms (Kong et al., 2018). This role may be vital for countries with emerging economies. In recent decades, emerging economies have mainly engaged in the production and processing of labor-intensive manufacturing industries, and the rapid growth of their economies and trade has relied heavily on labor. However, the demographic dividend in many emerging economies is gradually disappearing due to population aging and other issues, leaving many enterprises with a dilemma of a labor shortage and rising labor costs. Therefore, it is critical for businesses in emerging economies to increase the effectiveness of labor investment, which is also the focus of our study.

The motivation for our study is to explore the factors that affect corporate labor investment efficiency (LIE) in emerging economies. The effectiveness with which businesses allocate labor as a resource is known as LIE. Compared to other material capital investments, labor investment has distinctive characteristics. Since a person with intellectual and physical vigor is the target of labor investment rather than a material object, the elements taken into account for the investment will be more complicated, and it may be more challenging to improve the efficiency to reach the ideal level of labor investment. Therefore, the studies that have been applied to the efficiency of material capital may not be applicable to the studies of corporate LIE.

Due to profound combination of digital technologies like the Internet, big data, AI, and blockchain with the financial business, digital finance (DF), as an emerging financial model, has seen rapid growth around the world in recent years. Taking China as an example, the DF index rose at an annual pace of 29.1% on average from 2011 to 2020, and the depth and coverage of DF are increasing (Guo et al., 2020). DF upends established financial business models by enabling financing, payments, investments, and other new sorts of financial services through the use of digital technology (Lee and Shin, 2018). Therefore, it is evident that DF has remodeled the existing financial ecology and altered firms’ investment and financing environment, so whether DF has an influence on the corporate LIE is a question to be explored.

The objective of our study is to investigate the influence of DF on corporate LIE. From a theoretical standpoint, the influence we investigate appears uncertain. On the one hand, DF escapes the shackles of conventional finance through data-driven and product innovation, enhances the efficiency of financial institutions in approving loans, alleviates financing constraints of firms (Ozili, 2018), and thus may improve the LIE. On the other hand, DF development facilitates the concealment of financial risks (Vallée and Zeng, 2019), providing opportunities for firms to circumvent regulation for arbitrage and encourages the financialization of firms (Li, 2019), all of which may have a negative influence on the LIE.

In light of the analysis above, we raise three key research questions: Does DF promote or inhibit the corporate LIE? How does DF influence LIE? And do the effects vary depending on the environment and circumstances?

To address these issues, we chose Chinese A-share listed businesses from 2011 to 2020 as our study sample and construct an empirical model to examine whether DF improves corporate LIE, followed by endogeneity tests and robustness tests. Then, we explore the path mechanisms by which DF affects the corporate LIE and whether the effects may vary under different environments and circumstances. Finally, we conclude and provide policy recommendations in light of our results.

Why do we pick China as the context for our study? The following are the causes: On the one hand, as a significant emerging economy, China’s economy is booming rapidly (Khan et al., 2022; Zahid et al., 2022) and significant market expansion potential. China’s DF is currently leading the world in terms of growth pace and application scale (Tang et al., 2020), but its business model also needs to be further improved. There are still many opportunities and challenges ahead for DF. On the other hand, China has the largest population around the world, and its large labor force creates favorable demographic conditions for rapid economic development (Wang and Luo, 2022). However, with the emergence of an aging population, China’s demographic dividend is gradually vanishing. By 2021, there are more than 200 million people aged 65 and above in China, making up nearly 14% of the total population, which means that China has officially entered the “aging society” (Song and Gao, 2022). The growing aging population is a significant challenge to workforce development. Therefore, it may be interesting and meaningful to conduct the following research in the context of China, such a vibrant and intricate emerging economy.

The following are some potential contributions of our work: Firstly, our study enriches the research on the elements affecting the LIE of firms. Prior studies on the influencing elements have mostly concentrated on the impacts of internal elements on LIE, while studies on external factors, particularly macroeconomic policies, are limited. In this research, we choose the perspective of DF in macroeconomic policies to analyze its real influence on the LIE to alleviate the problems of labor scarcity and rising labor costs in emerging economies due to population aging. Secondly, our work adds to the body of knowledge about the economic consequences of DF. In recent years, the macroeconomic effects of DF and its microeconomic influence on businesses have been the subject of much academic research, yet labor, as an important element of firms, has not received the attention it deserves from the impact of DF. In our research, we dialectically analyze the positive and negative aspects of DF and investigate the effects of multiple dimensions of it on the corporate LIE, which offers recommendations to promote the healthy growth of DF. Thirdly, we further discuss the paths through which DF promotes LIE. We notice that the growth of DF may alleviate firms’ financing constraints and optimize their human capital structure, both of which have a beneficial impact on LIE. Such a pathway study opens the “black box” of how the growth of DF affects the LIE. Last but not least, we also investigate the boundary conditions of the influence of DF on LIE, which can better promote the LIE of enterprises in economically backward regions and of private nature. Our research offers empirical evidences for future regional economic growth as well as policy support for enterprises of different nature.

The remainder of this paper is arranged as shown below. The hypotheses are presented in section “Literature review and research hypothesis” after a review of the relevant literature. The models, variables, and data are introduced section “Sample and empirical methodology.” The findings are presented in section “Empirical results.” The influencing mechanism testing and heterogeneity analysis are presented in section “Additional analyses.” Section “Conclusions and policy recommendations” concludes.

## Literature review and research hypothesis

### Literature review

Judging from the existing literature, academic research on the economic consequences of DF mainly focus on the following aspects: The first is to concentrate on the macroeconomic effects of DF development, such as DF promoting economic development (Ahmad et al., 2021), narrowing the urban-rural wealth disparity (Bittencourt, 2006), helping impoverished areas get rid of poverty (Lacalle-Calderon et al., 2018), and promoting the overall social employment and entrepreneurship (Beck et al., 2018); Second, at the micro enterprise level, DF alleviates the financing constraints by lowering financing costs (Laeven et al., 2015), improves the scientific and technological innovation of firms (Beck et al., 2018; Sun, 2020), optimizes the human capital structure of enterprises (Jin and Yu, 2021), promotes the upgrading of industrial structure (Demertzis et al., 2018); Additionally, DF also has an impact on management innovation and product innovation of conventional financial organizations (Norden et al., 2014; Lorente et al., 2018); The third is to investigate the risks and adverse effects embedded in the growth of DF. Its growth encourages corporate financial investment, and the phenomenon of enterprises “departing from the real to the virtual” progressively emerges (Du et al., 2019). DF has the potential to amplify underlying risks while also altering the norm of financial distribution, making pre-existing risks more insidious and complicated (Vallée and Zeng, 2019).

According to the existing literatures, the factors impacting LIE are mostly focused on the following aspects: From the standpoint of information quality, high-quality financial reports (Jung et al., 2019b), environmental information disclosure (Jiang et al., 2020), and informative stock prices (Ben-Nasr and Alshwer, 2016) can all help to increase LIE; From the standpoint of internal governance, CEO characteristics (Khedmati et al., 2020), corporate strategy (Habib and Hasan, 2021) and corporate governance (Oh and Park, 2022) all have an influence on LIE; As for external monitoring, investors’ attention (Ghaly et al., 2020) and stock analysts’ follow-up attention (Chen et al., 2018) are significantly and positively correlated with LIE; In terms of the environment in which they are located, the promotion of politicians in the location of the firm (Kong et al., 2020) and the uncertainty of the environment (Jun and Sun, 2020) can reduce the LIE, while digital economy (Zhai et al., 2022), environmental regulation (Chen et al., 2022) and external labor market incentives (Chowdhury et al., 2022) can improve it. Furthermore, labor protection (Koeniger and Leonardi, 2007) has an influence on the corporate LIE through changing their labor costs.

Through a study of the literature, we discover that: the literature on the inclusive nature of DF is relatively well established, and many studies have shown that DF has been helpful in the overall enhancement of the macroeconomy, the transformation of conventional financial organizations, and the financing and innovation of enterprises. What also merits our attention is the fact that several academics have highlighted the risks and drawbacks in the current growth of DF. Therefore, despite many benefits of the flourishing DF, we shouldn’t neglect these drawbacks in the upcoming theoretical analysis procedure. Turning to the LIE, there is a dearth of study, and the existing studies mainly concentrate on the information quality, corporate governance, and external regulation and so on. Labor investments are as significant and more complicated than other types of material capital investments made by firms, and therefore deserve greater consideration. Therefore, our paper reevaluates the effects of DF and investigates whether it can boost LIE, which can both enrich pertinent research in the area of corporate LIE and give theoretical support for healthy growth of DF.

### Research hypothesis

Based on the existing literature, our research indicates that the influence of DF on the LIE may include the following aspects:

On the one hand, based on pecking financial order theory and endogenous financial development theory, we contend that DF might improve corporate LIE by alleviating financing constraints and optimizing the structure of human capital. The specific analysis is as follows: Because the labor costs may be composed of both variable and fixed costs (Hamermesh, 1993), the process of labor investment involves costs adjustments and the need for financing. Pecking financial order theory states that companies would prioritize financing with internal funds, and that if internal funds are insufficient, companies may be forced to utilize external capital, such as external bonds or external equity, to finance their labor investments (Ben-Nasr and Alshwer, 2016). Due to information asymmetry between corporations and banks and corporate moral hazard, the previous traditional financing processes were difficult and expensive to evaluate. As a result, financing for companies became readily constricted, which in turn may have decreased the effectiveness of labor investment (Deng and Zhao, 2022). Based on endogenous financial development theory, Ren (2020) and Majeed et al. (2022) believes that DF, as a fresh business model for the combination of finance and technology, may compensate for the inefficiencies and “mismatch” of conventional finance, thereby may alleviating financing constraints of enterprises.

Specifically, there are three possible paths for DF to alleviate corporate financing constraints: Firstly, DF applies digital technology to innovate traditional financial services and products (Cheng and Wang, 2022), lowing the threshold of financial services, broadening financing channels, and expanding financing scale so that more enterprises may benefit from financing (Ahmad et al., 2021). Secondly, the issue of information asymmetry between financial organizations and non-financial businesses could be efficiently resolved through DF. (Demertzis et al., 2018). Based on big data, DF deeply digs and analyzes firms’ information, and establishes an independent credit investigation system to assist financial institutions grasping a corporation’s operating capacity and financial status in a timely manner. This alleviates the concerns of financial institutions about the opaque information and high lending risks posed by enterprises, and increases their willingness to lend to enterprises. Thirdly, DF relies on the digital credit system to overcome geographical limitations, simplify the credit approval process and accelerate credit approval, all of which improve the efficiency of corporate financing. Thus, it is obvious that DF has a positive influence on the channels, scale and speed of financing, which in turn alleviates the financing constraints (Laeven et al., 2015). DF alleviates financing constraints for enterprises, facilitating the acquisition of financing to fill the labor shortfall required for ongoing operations in the context of labor under-investment (Ferreira et al., 2011), which in turn may improve the LIE.

Furthermore, DF might improve corporate LIE by optimizing human capital structure. Firstly, the growth of DF drives the R&D investment and technological innovation of companies (Yang et al., 2021). In order to perform R&D and innovation, enterprises need to bring in senior talents with high level of education or extensive skills and experience, therefore optimizing the human capital structure (Li and Liu, 2018). Secondly, the development of DF also encourages enterprises to undergo digital transformation, making them more intelligent and automated in their production and operation processes. Many easy and repetitive manual jobs are now replaced by intelligent program operations, which lowers personnel redundancy and optimizes the human capital structure. Thirdly, as DF has advanced, financial institutions’ digital payment and education-specific deposit businesses have improved, providing convenience and financial support for enterprises’ working employees to pursue their education (Li, 2020). In recent years, a rising number employees have pursued MBA, MPA, MEM and other on-the-job degrees after work through various ways such as distance education and online-offline combination, leading to an increase in the proportion of on-the-job postgraduates in China. This phenomenon also optimizes the human capital structure of firms. People are the main body of labor, and the optimization of human capital structure increases the labor productivity of enterprises, which may improve firms’ LIE.

The foregoing analysis leads to the following Hypothesis 1a:

**H1a:** Digital finance improves corporate LIE.

On the other hand, DF could inhibit corporate LIE. First of all, DF reshapes the current financial ecosystem and alters the investment and financing climate of firms (Sun and Shen, 2021). According to investment substitution theory, when the rate of return on financial investments is higher than the rate of return on real investments, many non-financial enterprises tend to stray from their main business and invest heavily in financial assets in order to increase their profits, resulting in the phenomenon of “departing from the real to the virtual.” More capital is being withdrawn from the main business, crowding out resources for fixed asset investment and technological innovation (Wang et al., 2017), leaving enterprises with insufficient funds for equipment upgrading, human resource improvement and labor replenishment, potentially inhibiting the LIE. Secondly, we explain that DF may alleviate firms’ financing constraints in the above. However, alleviating financing constraints may also inhibit the LIE in some firms. Firms that over-invest in labor, with the help of external finance, may forgo implementing policies to streamline employees and spend more money on labor investment, which may result in staff redundancy and thus reduce corporate LIE. Thirdly, there is also the possibility that DF cannot alleviate corporate financing constraints at the root. As a combination of technological innovation and traditional finance, the essence of DF is still finance, which means that financial risks are unavoidable in its functioning. Meanwhile, as DF develops, the boundaries between regions and financial institutions become increasingly blurred (Chen Y. et al., 2021), making financial risks more hidden and complex as well (Tang et al., 2020). Coupled with the regulatory lag caused by the imperfect system (Li, 2019), it is difficult for enterprises to screen effective financing proposals (Lee and Shin, 2018). It may not be able to alleviate the corporate financing constraints at the root, causing difficulties in their operation, which is not conducive to the corporate LIE.

Hypothesis 1b is proposed based on the foregoing analysis:

**H1b:** Digital finance inhibits corporate LIE.

## Sample and empirical methodology

### Sample selection

Considering the availability of the Digital Inclusive Finance Index, we select Chinese A-share listed businesses from 2011 to 2020 as the preliminary sample and treat them as follows to improve the reliability of the results: (1) Financial industry firms are excluded. (2) Firms with incomplete data are excluded. (3) Firms with special treatment are excluded. (4) Winsorizing is done on all continuous variables between the first and 99th percentiles. The final sample of 3465 firms after screening contains 23,503 observations. The data for the China Digital Inclusive Finance Index are sourced from the Digital Finance Research Centre of Peking University, while the remainders come from the CSMAR database.^1^

### Definition of variables

#### Dependent variable – Labor investment efficiency

The term “LIE” describes how far the actual value of a labor investment deviates from its ideal value. According to Pinnuck and Lillis (2007), we utilize the rate of turnover in the number of employees to indicate the change in the number of employees in each firm. Specifically, we use the necessary financial indicators to anticipate the ideal employee turnover rate, then subtract the ideal employee turnover rate from the actual rate, and the resulting difference can be used to measure the inefficient investment in labor. Referring to Jung et al. (2019a), the formula for calculating the LIE is as follows.


$$N\,E\,T\,\_\,H\,I\,R\,E_{i,t}=\beta_{0}+\beta_{1}\,S\,A\,L\,E\,S\,G\,R\,O\,W\,T\,H_{i,t-1}$$

$$+\beta_{2}\,S\,A\,L\,E\,S\,G\,R\,O\,W\,T\,H_{i,t}+\beta_{3}\,\Delta \,R\,O\,A_{i,t-1}$$

$$+\beta_{4}\,\Delta \,R\,O\,A_{i,t}+\beta_{5}\,R\,O\,A_{i,t}+\beta_{6}\,R\,E\,T\,U\,R\,N_{i,t}$$

$$+\beta_{7}\,{\text{SIZE}}_{i,t-1}+\beta_{8}\,\Delta \,Q\,U\,C\,I\,K_{i,t-1}$$

$$+\beta_{9}\,\Delta \,Q\,U\,C\,I\,K_{i,t}+\beta_{10}\,Q\,U\,C\,I\,K_{i,t-1}$$

$$+\beta_{11}\,{\text{LEV}}_{i,t-1}+\beta_{12}\,{\mathrm{LOSSBIN1}}_{i,t-1}$$

$$+\beta_{13}\,{\mathrm{LOSSBIN2}}_{i,t-1}+\beta_{14}\,{\mathrm{LOSSBIN3}}_{i,t-1}$$

$$+\beta_{15}\,{\mathrm{LOSSBIN4}}_{i,t-1}+\beta_{16}\,{\mathrm{LOSSBIN5}}_{i,t-1}$$

(1) $$+\varepsilon_{i,t}$$

Where i and t stands for a firm and a year, respectively. SALESGROWTH denotes the growth rate of sales, ROA denotes the return on assets, ΔROA denotes the change in ROA, RETURN denotes the annual stock return, SIZE is calculated as the natural logarithm of market value, QUICK denotes the quick ratio; ΔQUICK indicates the change in QUICK; LEV indicates the leverage ratio. Each lossbin is a dummy variable, with five lossbins representing each 0.005 interval of ROA in year t−1, ranging from 0 to −0.025. If previous-year ROA is from −0.005 to 0, Lossbin1 equals 1, else it equals zero. If previous-year ROA is from −0.01 to −0.005, Lossbin2 equals 1, else it equals zero. Lossbin3, Lossbin4 and Lossbin5 have similar definitions. ε denotes residuals.

The LIE is obtained by calculating the absolute value of the residual after regression with year and industry effects controlled. LIE is an inverse indicator. A larger value indicates that the net employment turnover rate deviates more from the ideal level and the lower the LIE. Specifically, when the residuals are positive, the actual net employment change rate is higher than the ideal level, indicating over-investment in labor; when the residuals are negative, the actual net employment change rate is lower than the ideal level, indicating under-investment in labor.

#### Independent variable – Digital Finance

The Digital Inclusive Finance Index (Guo et al., 2020) released by the Digital Finance Research Center of Peking University has been widely utilized in Chinese digital finance research. This Index set includes city-level indices for the years 2011 to 2020, which not only measures DF in general, but also provides detailed DF indices of sub-dimensions like breadth of coverage and depth of use. Given its authority and widespread use, the index is also utilized to measure DF in our study. DF is a macro variable at the city level, while this paper intends to explore its influence on micro firms, so we determine the city where each company is located, and then the Digital Inclusive Finance Index for that city is the Digital Inclusive Finance Index that corresponds to the company. To ensure that the index is not too large compared to other variables, we divide the Digital Inclusive Finance Index by 1000. In addition, we supplement two main sub-dimensions of DF, Breadth of coverage (Breadth) and Depth of use (Depth), as sub-index to test our hypothesis (Wu et al., 2020).

#### Control variables

In order to mitigate the impact of omitted variables, we control for multiple variables at the regional, industry and firm levels. At the regional level, a city’s labor force level often exhibits variations depending on the quality of local economic growth, so referring to Chen J. et al. (2021), Lang et al. (2021), and Li et al. (2022) we account for the economic development level (GDP) of the city where the company is located. At the industry level, referring to Wang and Sun (2018), we control for degree of industry monopoly (ILI), companies with monopolistic power often have the capacity to rely on higher product pricing to generate surplus profits, which enables them to have enough money to invest in capital, labor, and other inputs of production. At the firm level, we choose control variables from three aspects: fundamental business characteristics, financial status and governance structure. First, the LIE could be impacted by the fundamental characteristics of the company. Referring to Jung et al. (2019b) and Guan (2021), We chose three company characteristics—age (Age), size (Assets) and labor intensity (LI). Specifically, companies with larger size have more complicated organizational structures and are more prone to have employees floating around, which may increase the possibility of inefficient labor investment. Next, we focus to the financial condition with reference to Jung et al. (2019b); Sheng and Li (2015), Ben-Nasr and Alshwer (2016), and Chu and Fang (2020) then control for the following typical financial indicators: leverage ratio (Lev), net fixed assets (FA), sales growth (Growth), current ratio (CR) and capital intensity (Capital). Specifically, companies with high capital intensity tend to use advanced production technologies and equipment, which directly determine labor productivity, and therefore this is likely to influence the corporate labor investment. Finally, from the standpoint of governance structure, different governance structures may lead to different hiring and firing regimes in a firm, which can also affect the LIE. Therefore, we control for the equity concentration (H10) and the integration of two positions (DUAL) with reference to Khedmati et al. (2020). Appendix Table 1 contains the definition for the above variables.

### Empirical model

Referring to Majeed et al. (2021) and Chishti and Sinha (2022), the following procedures are performed to conduct the empirical study: multicollinearity test, unit root test, cointegration test, Hausman test and selection of regression model.

#### Multicollinearity test

Multicollinearity refers to the difficulty of estimating results from linear regression models due to the presence of exact or high correlations between explanatory variables. We choose the variance inflation factor (VIF) test to check whether any variables have issues with multicollinearity.


$$V\,I\,F=\frac{1}{1-R_{i}^{2}}$$

$R_{i}^{2}$ is the coefficient of determination derived by fitting the ith variable as the dependent variable to other independent variables. A higher VIF value denotes a stronger correlation between the variable and other independent variables. The regression model suffers severe multicollinearity if the VIF is more than 10 in general. Table 1 displays the VIF test results for the variables utilized in this investigation. All of the variables’ VIF values are below 10, which shows that multicollinearity is not a concern for them.

**TABLE 1:** Multicollinearity test.

Variables	VIF	Variables	VIF
DF	1.34	Growth	1.05
GDP	1.27	CR	1.91
ILI	1.12	LI	1.29
Age	1.10	Capital	1.29
Assets	2.92	H10	1.12
Lev	2.03	DUAL	1.06
FA	2.55		

#### Heteroscedasticity tests

To check for heteroskedasticity in the regression model, we conduct both the Breusch–Pagan test and the White test. Their outcomes are displayed in Table 2. The original hypothesis H0 for both tests is that heteroskedasticity does not exist in the model, while the *p*-value in the results is 0.0000, which strictly rejects the original hypothesis and suggests the existence of heteroskedasticity in the model.

**TABLE 2:** Heteroskedasticity tests.

Methods	chi2	*P*-value
Breusch-Pagan test	7295.11	0.0000
White test	1029.83	0.0000

#### Unit root tests

To verify the smoothness of the data and avoid pseudo-regressions, we perform the unit root test. Unit root testing can be performed using a variety of methods, and the outcomes may occasionally differ depending on the method used. To enhance the robustness of the results and take into account the fact that the data in our paper are unbalanced panel data, we choose two tests, the IPS test and Fisher-ADF test. For both tests, the original hypothesis H0 is that the variables don’t have a unit root. Table 3 displays the specific test outcomes.

**TABLE 3:** Unit root tests.

Variables	IPS	Fisher-ADF	Variables	IPS	Fisher-ADF
LIE	–38.0910*** (0.0000)	82.1575*** (0.0000)	d_LIE	–43.5899*** (0.0000)	118.7799*** (0.0000)
DF	–27.7946*** (0.0000)	19.1744*** (0.0000)	d_DF	–35.3110*** (0.0000)	66.1070*** (0.0000)
GDP	–14.4705*** (0.0000)	–15.9972 (1.0000)	d_GDP	–11.0541*** (0.0000)	3.8164*** (0.0001)
ILI	–23.7164*** (0.0000)	9.9819*** (0.0000)	d_ILI	–30.0332*** (0.0000)	30.9419*** (0.0000)
Age	–22.7135*** (0.0000)	–15.9481 (1.0000)	d_Age	–32.4136*** (0.0000)	–24.8143*** (0.0000)
Assets	–25.2025*** (0.0000)	25.7907*** (0.0000)	d_Assets	–37.9150*** (0.0000)	50.4165*** (0.0000)
Lev	–13.8729*** (0.0000)	21.3896*** (0.0000)	d_Lev	–32.8982*** (0.0000)	53.9126*** (0.0000)
FA	–10.0567*** (0.0000)	47.1195*** (0.0000)	d_FA	–30.4969*** (0.0000)	65.1182*** (0.0000)
Growth	–32.6131*** (0.0000)	42.6861*** (0.0000)	d_Growth	–38.9570*** (0.0000)	79.1805*** (0.0000)
CR	–19.5884*** (0.0000)	31.3821*** (0.0000)	d_CR	–35.4416*** (0.0000)	56.9160*** (0.0000)
LI	–27.6547*** (0.0000)	64.6265*** (0.0000)	d_LI	–39.6352*** (0.0000)	74.1179*** (0.0000)
Capital	–12.0904*** (0.0000)	18.2377*** (0.0000)	d_Capital	–29.2972*** (0.0000)	41.9358*** (0.0000)
H10	–13.6279*** (0.0000)	33.9985*** (0.0000)	d_H10	–32.4660*** (0.0000)	80.2568*** (0.0000)
DUAL	–0.8805 (0.1893)	9.4534*** (0.0000)	d_DUAL	–35.0924*** (0.0000)	32.5774*** (0.0000)

(1) d_ denotes the first-order difference. (2) ***, **, and * Denote statistical significance at the 1, 5, and 10% levels, respectively.

As noted in Table 3, there are a few variables with *P*-values greater than 0.01 under different tests, such as Age and DUAL, indicating that that these variables have a unit root and that the outcomes for these variables cannot reject the original hypothesis. However, after running the first-order difference, the *P*-values of the variables are all less than 0.01, and this test outcome rejects the original hypothesis. This shows that our difference variables are smooth and there is no unit root for any of the difference variables. Additionally, this finding suggests that our variables may have a stable long-term cointegration relationship, therefore additional research is required to validate this.

#### Cointegration test

To test whether our variables have a stable cointegration relationship over time, we perform a cointegration test. We choose for the Kao test since we have a lot of variables—more than seven. The results in Table 4 show that the *p*-values of Modified Dickey-Fuller (MDF), Dickey-Fuller (DF), and Augmented Dickey-Fuller (ADF) are less than 0.01. These results reject the original hypothesis, which is that there is no cointegration relationship. Therefore, our finding suggests there is a cointegration relationship between variables.

**TABLE 4:** Cointegration test.

Method	Type	Statistic	*P*-value
Kao	MDF	–29.6914	0.0000
	DF	–66.7869	0.0000
	ADF	–29.1790	0.0000

Based on the tests mentioned above, we find that there is a cointegration relationship between variables in our paper. Additionally, there is heteroskedasticity but no multicollinearity or unit root between the variables, which indicates that we should consider the heteroskedasticity problem in the next model selection.

#### Regression model

Referring to the majority of existing literature (Jung et al., 2019b; Ben-Nasr and Alshwer, 2016; Jiang et al., 2020), we adopt a least squares model (OLS) for regression to explore the influence of DF on the corporate LIE. Additionally, taking into account the existence of heteroskedasticity in the aforementioned test results, we estimate to eliminate heteroscedasticity using robust standard errors in the model.


(2) $$L\,I\,E_{i,t}=\alpha_{0}+\alpha_{1}\,D\,F_{i,t}+\sum_{j}\alpha_{j}\,C\,o\,n\,t\,r\,o\,l_{j,i,t}+\varepsilon_{i,t}$$

Where i, t and j denote a firm, a year and a control variable, respectively. ε denotes residuals. LIE denotes for the corporate LIE as the dependent variable, DF for digital finance as the independent variable, and Control for the collection of control variables. In order to minimize the impacts of other factors on LIE, such as the region and industry to which the firm belongs during the observation period, we also control for year (Year), industry (Industry) and province (Region).

In addition, to guarantee the robustness of the outcomes, we decide to choose a model to replace OLS model for the regressions in the ensuing robustness tests. For this purpose, we run the Hausman test, which yields a chi-square statistic of 318.20, equating to a *p*-value of 0.0000, so we choose the fixed effects model rather than the random effects model for the robustness test.

## Empirical results

### Descriptive statistics

Descriptive statistics for key variables are reported in Table 5. The mean value of LIE is 0.189, with maximum and minimum values of 2.406 and 0, respectively, suggesting that firms generally suffer from LIE. The LIE is further decomposed into over-investment in labor and under-investment in labor, the data in the table shows that there are 16,127 sample observations with residuals less than 0, accounting for 68.62% of the total. This outcome is in line with the findings of Kong et al. (2020), indicating that most firms in China’s A-share market have insufficient labor investment, which validates our previous analysis that Chinese firms are gradually falling into the dilemma of labor shortage and rising labor costs.

**TABLE 5:** Descriptive statistics.

Variables	*N*	Mean	*SD*	Min	Max
LIE	23503	0.189	0.312	0.000	2.406
Over-investment in labor	7376	0.307	0.554	0.000	2.406
Under-investment in labor	16127	–0.140	0.120	–1.131	–0.000
DF	23503	0.220	0.070	0.057	0.322
Breadth	23503	0.219	0.067	0.004	0.326
Depth	23503	0.217	0.075	0.013	0.350
FC	23503	0.463	0.280	0.005	0.933
HT	23503	3.126	0.0529	0.000	0.659
GDP	23503	10.506	0.714	8.061	11.615
ILI	23503	0.119	0.072	0.012	0.378
Age	23503	22.801	5.507	6	121
Assets	23503	21.924	1.195	19.540	25.593
Lev	23503	0.439	0.206	0.059	0.906
FA	23503	20.321	1.700	15.539	24.793
Growth	23503	0.155	0.411	–0.592	2.607
CR	23503	2.253	2.156	0.296	14.088
LI	23503	0.095	0.087	0.003	0.490
Capital	23503	2.643	2.375	0.405	16.031
H10	23503	57.196	15.09	22.590	90.110
DUAL	23503	0.255	0.436	0	1

### Results and analysis

#### Baseline results

Table 6 summarizes the outcomes in model (2). In Column (1), the regression coefficient of DF without accounting for other variables exhibits a significantly negative correlation with LIE at the 1% level. And even after controlling for variables at the firm level and in Columns (2), as well as variables at the firm level, region, and industry three levels in Columns (3), the regression coefficient of DF is still significantly negatively connected with LIE at the 1% level. Since LIE is an inverse indicator, with larger values indicating less efficient labor investment, the results, which are in line with the study of Zhai et al. (2022), suggest that the growth of DF can enhance LIE, supporting hypothesis H1a.

**TABLE 6:** Baseline results.

	(1)	(2)	(3)
	LIE	LIE	LIE
DF	–0.476*** (–15.10)	–0.398*** (–12.24)	–0.191*** (–2.58)
Age		0.002*** (4.21)	0.002*** (4.31)
Asset		–0.039*** (–9.30)	–0.039*** (–9.30)
Lev		0.068*** (4.49)	0.064*** (4.18)
FA		0.013*** (4.34)	0.013*** (4.34)
Growth		0.232*** (19.57)	0.232*** (19.49)
CR		–0.003*** (–2.86)	–0.003*** (–3.02)
LI		–0.286*** (–10.26)	–0.287*** (–10.28)
Capital		0.013*** (9.92)	0.013*** (9.97)
H10		0.001*** (5.20)	0.001*** (5.14)
DUAL		0.003 (0.72)	0.003 (0.74)
GDP			–0.06*** (–2.87)
ILI			–0.099*** (–3.00)
Constant	0.361*** (17.05)	0.802*** (14.70)	1.375*** (6.57)
Year	Yes	Yes	Yes
Industry	Yes	Yes	Yes
Region	Yes	Yes	Yes
Adj.*R*^2^	0.020	0.136	0.137
*N*	23503	23503	23503

***, **, and * Denote statistical significance at the 1, 5, and 10% levels, respectively. T-statistics are based on robust standard errors and are presented in parentheses.

#### Sub-index regression results

We conduct a supplementary test using the sub-dimensions of DF, the breadth of coverage (Breadth) and the depth of use (Depth) as explanatory variables, and the outcomes are listed in Table 7, where the regression coefficient of both Breadth and Depth of DF are significantly negative correlation with LIE at the 1% level. According to the results, which are in line with those of the baseline regression, both the breadth of coverage and the depth of use of digital inclusive financing might improve corporate LIE. This demonstrates that the enhancement of LIE through DF is due to both the increasing coverage group and the continuous upgrading and optimization of digital products and services.

**TABLE 7:** Sub-index regression results.

	(1)	(2)	(3)	(4)	(5)	(6)
	LIE	LIE	LIE	LIE	LIE	LIE
Breadth	–0.465*** (–14.36)	–0.374*** (–11.25)	–0.274*** (–7.80)			
Depth				–0.473*** (–15.39)	–0.416*** (–13.19)	–0.403*** (–5.85)
Age		0.002*** (4.28)	0.002*** (4.99)		0.002*** (4.20)	0.002*** (4.28)
Asset		–0.04*** (–9.55)	–0.038*** (–9.15)		–0.038*** (–9.12)	–0.038*** (–9.12)
Lev		0.072*** (4.74)	0.063*** (4.18)		0.066*** (4.33)	0.062*** (4.05)
FA		0.013*** (4.45)	0.012*** (3.91)		0.013*** (4.23)	0.013*** (4.23)
Growth		0.232*** (19.54)	0.233*** (19.68)		0.233*** (19.66)	0.233*** (19.57)
CR		–0.003*** (–2.65)	–0.003** (–2.50)		–0.003*** (–2.97)	–0.003*** (–3.07)
LI		–0.281*** (–10.11)	–0.276*** (–10.12)		–0.290*** (–10.37)	–0.289*** (–10.32)
Capital		0.013*** (9.88)	0.013*** (9.89)		0.013*** (9.91)	0.014*** (10.01)
H10		0.001*** (5.24)	0.001*** (5.64)		0.001*** (5.19)	0.001*** (5.23)
DUAL		0.003*** (0.63)	0.005 (1.06)		0.003 (0.74)	0.003 (0.74)
GDP			–0.006* (–1.79)			–0.000 (–0.00)
ILI			–0.142*** (–4.37)			–0.100*** (–3.02)
Constant	0.358*** (16.87)	0.81*** (14.83)	0.802*** (13.27)	0.359*** (17.04)	0.797*** (14.61)	0.802*** (4.02)
Year	Yes	Yes	Yes	Yes	Yes	Yes
Industry	Yes	Yes	Yes	Yes	Yes	Yes
Region	Yes	Yes	Yes	Yes	Yes	Yes
Adj.*R*^2^	0.019	0.135	0.131	0.021	0.137	0.138
N	23503	23503	23503	23503	23503	23503

***, ** and * Denote statistical significance at the 1, 5, and 10% levels, respectively. T-statistics are based on robust standard errors and are presented in parentheses.

### Robustness tests

#### Endogeneity test

We employ the instrumental variable method to test the endogeneity of the model. The instrumental variable (Major) we choose, which is a dummy variable, is whether the major of the city mayor is related to DF (Jin and Yu, 2021). This decision is being made for two primary reasons: Firstly, while formulating future urban development plans, mayors may be more inclined to focus on their areas of expertise. Therefore, whether the major of the mayor is related to DF may have a certain influence on city’s DF growth and meet the correlation assumption of instrumental variables. Secondly, when the mayor he picked his major, he had no idea whether he would become a mayor in the future, he just chose depending on his interests and hobbies when he was an undergraduate or graduate student. Therefore, this instrumental variable is typical historical data. In addition, individual behaviors are unlikely to have a significant influence on the city level, satisfying the exogenous assumption of instrumental variables. When collating the data, we set the instrumental variable to 1 if the mayor’s major is one of theoretical economics, applied economics, technical economics or information and communication, all of which are relevant to DF, otherwise it is 0. The data sources of the instrumental variable are mainly composed of two parts. The data for 2011–2016 are mainly from the database of “Provincial and Municipal Leaders Information,” the data from 2017 onwards are primarily collated by the authors through searching the official websites of various provinces and cities, Leadership Database, Baidu encyclopedia and other platforms. After our manual sorting and collation, mayors with majors in DF accounted for about 39.93% of the total observations, which is consistent with the findings of Jin and Yu (2021).

Table 8 shows the outcomes of instrumental variables tests. In column (1), the results of the first stage indicates that Major is significantly positively correlated with DF. In column (2), the second stage outcomes indicate that the regression coefficient of DF is significantly negatively correlated with LIE at the 1% level. This suggests that, after we use the instrumental variable tests to deal with endogeneity, DF may still significantly improve the LIE. Furthermore, the Kleibergen-Paap rk Wald *F* statistic is 910.063, while the corresponding critical value at the 10% level of the Stock-Yogo test is 16.38, the Kleibergen-Paap rk Wald *F* statistic is greater than the value at the 10% level of the Stock-Yogo test, proving that the weak identification test is passed.

**TABLE 8:** Instrumental variables tests.

	First-stage	Second-stage
	
	(1)	(2)
	DF	LIE
Major	–0.024*** (–30.17)	
DF		–1.071*** (–6.24)
Constant	–0.395*** (–37.07)	0.798*** (8.95)
Control	Yes	Yes
Industry	Yes	Yes
Year	Yes	Yes
Adj.*R*^2^	0.2785	0.017
*N*	23503	23503

(1) ***, **, and * Denote statistical significance at the 1, 5, and 10% levels, respectively. T-statistics are based on robust standard errors and are presented in parentheses. (2) Kleibergen-Paap rk Wald F statistic is 910.063, and the corresponding critical value at the 10% level of the Stock-Yogo test is 16.38, indicating that it passed the weak identification test.

#### Other robustness test

To guarantee the robustness of the outcomes, the robustness tests are performed in the following way: Firstly, we substitute the baseline OLS model with the fixed effects model that controls for the effects of firm and time, and the outcomes are displayed in columns (1)–(3) of Table 9. Secondly, since municipalities directly under the Central Government have significant economic and political particularities, the degree of DF and corporate LIE in these municipalities may differ greatly from that of other cities. So, after dropping the samples of municipalities directly under the central government, we re-run the regression, and the outcomes are displayed in columns (4)–(6) of Table 9. Thirdly, considering the significant influence caused by COVID-19 in 2020 (Wu and Zhu, 2021; Ma et al., 2022), we also re-run the regression test after excluding the 2020 sample, with the results displayed in columns (7)–(9) of Table 9. The outcomes of the above test suggest that the core conclusion of our research, “the growth of DF can improve the LIE” still holds.

**TABLE 9:** Other robustness tests.

	Two-way fixed effects			Exclude municipalities			Excluding the impact of the new crown epidemic		
	(1)	(2)	(3)	(4)	(5)	(6)	(7)	(8)	(9)
	LIE	LIE	LIE	LIE	LIE	LIE	LIE	LIE	LIE
DF	–0.226** (–2.09)			–0.365*** (–11.35)			–0.405*** (–10.67)		
Breadth		–0.189* (–1.71)			–0.334** (–10.31)			–0.374*** (–9.49)	
Depth			–0.237** (–2.52			–0.390*** (–12.33)			–0.428*** (–11.81)
Constant	2.014*** (5.59)	2.159*** (6.29)	1.959*** (5.79)	0.764*** (13.15)	0.764*** (13.15)	0.755*** (13.01)	0.832*** (13.24)	0.841*** (13.39)	0.824*** (13.13)
Control	Yes	Yes	Yes	Yes	Yes	Yes	Yes	Yes	Yes
Firm	Yes	Yes	Yes	Yes	Yes	Yes	Yes	Yes	Yes
Year	Yes	Yes	Yes	Yes	Yes	Yes	Yes	Yes	Yes
Adj.R^2^	0.069	0.069	0.070	0.125	0.125	0.128	0.133	0.132	0.134
N	23503	23503	23503	18209	18209	18209	20279	20279	20279

***, **, and * Denote statistical significance at the 1, 5, and 10% levels, respectively. T-statistics are based on robust standard errors and are presented in parentheses.

## Additional analyses

### Influencing mechanism

The studies above suggest that DF development may promote corporate LIE, but the specific impact mechanism still remains at the level of theoretical analysis and lacks relevant empirical support. The above theoretical analysis points out that DF alleviates financing constraints and optimizes the human capital structure of firms, thereby improving corporate LIE. Therefore, we perform further empirical tests along the two influence paths of “DF-financing constraints- LIE” and “DF-human capital structure- LIE.”

In accordance with Fee et al. (2009), we utilize the FC index^2^ to gauge the severity of corporate financing constraints (FC). The severity of the financing constraints increases with FC size. In accordance with Yang and Kong (2019) and Zhai et al. (2022), we utilize the proportion of employees with master’s degree or higher compared the overall amount of employees in the firm to calculate the human capital structure (HT).

Following Baron and Kenny (1986), we run the following model:


(3) $$M\,e\,d\,i\,a\,t\,e_{k,i,t}=\lambda_{0}+\lambda_{1}\,D\,F_{i,t}+\Sigma_{j}\,\lambda_{j}\,C\,o\,n\,t\,r\,o\,l_{j,i,t}+\varepsilon_{i,t}$$

$$L\,I\,E_{i,t}=\mu_{0}+\mu_{1}\,M\,e\,d\,i\,a\,t\,e_{k,i,t}+\mu_{2}\,D\,F_{i,t}$$

(4) $$\;+\Sigma_{j}\,\mu_{j}\,C\,o\,n\,t\,r\,o\,l_{j,i,t}+\varepsilon_{i,t}$$

Where i, t, j and k denote a firm, a year, a control variable and an intermediate variable respectively. ε denotes residuals. Mediate denotes FC and HT. DF, LIE and Controls are in keeping with how the variables were defined in the previous baseline regression. We estimate to eliminate heteroscedasticity using robust standard errors in the model.

The model outcomes of the influencing mechanism are displayed in Table 10. From columns (1) and (2), we can see that DF is significantly negatively correlated with the FC, while FC and LIE are significantly positively correlated, which indicating that DF may improve corporate LIE by alleviating financing constraints. Similarly, we can see from columns (3) and (4) that DF has a significant positive correlation with the HT, while HT is significant positive correlation with LIE, which indicating that DF may improve corporate LIE by optimizing human capital structure. This result preliminarily verifies the influence paths of financing constraints and human capital structure, which is consistent with our theoretical analysis.

**TABLE 10:** Influencing mechanism tests.

	Financial constraints Mediate = FC		High Talent Mediate = HT	
	(1)	(2)	(3)	(4)
	Mediate	LIE	Mediate	LIE
DF	–0.088*** (–8.70)	–0.404*** (–13.89)	13.598*** (28.52)	–0.443*** (–15.16)
Mediate		0.154*** (8.26)		–0.001** (–2.09)
Constant	–3.146*** (–617.13)	0.674*** (11.12)	11.972*** (27.64)	0.367*** (13.83)
Control	Yes	Yes	Yes	Yes
Year	Yes	Yes	Yes	Yes
Industry	Yes	Yes	Yes	Yes
Region	Yes	Yes	Yes	Yes
Adj.*R*^2^	0.802	0.015	0.162	0.126
*N*	23503	23503	23503	23503

***, **, and * Denote statistical significance at the 1, 5, and 10% levels, respectively. T-statistics are based on robust standard errors and are presented in parentheses.

We also conduct a Bootstrap test to check the credibility of the results, we used Bootstrapping to conduct 1000 samples, and the results are shown in Appendix Table 2. The indirect mediation effects of financing constraints and human capital structure are −0.046 and −0.071, respectively. LLCI = −0.019 ULCI = −0.008 and LLCI = −0.022 ULCI = −0.000 on the 95% confidence interval, both of which do not include 0, suggesting that the indirect effect of mediation is significant at the 95% level. Therefore, we believe that DF helps companies alleviate the financing constraints and improve the human capital structure, which in turn increases their LIE.

### Heterogeneity analysis

Due to the fact that economic growth, resource endowment, and technology advantages differ between cities in China, we hypothesize that the impact of DF on LIE may be influenced by regional heterogeneity (Lei et al., 2021). Additionally, the form of property rights typically has a substantial impact on shaping the growth of firms under the Chinese institutional system (Ma and Zhu, 2022). Therefore, in this part, we analyze if there are variations in the influence of DF on LIE among enterprises in different regions and with different property rights.

Firstly, we separate China’s 31 provinces into three categories based on their geographical locations: east, central and west, and then run group regression. The outcomes are displayed in columns (1)–(3) of Table 11. DF has a greater positive impact on the LIE of companies in Chinese central and western region, which are characterized by relatively backward economies compared to the east. This indicates that, unlike most of the previous literature on economic regional heterogeneity, our finding does not follow the “Matthew effect,” in which “the strongest gaining stronger and the weakest going weaker.” On the contrary, the growth of DF could narrow the gap of LIE between regions, which provides a good opportunity for the balanced development of human capital in different regions. In this regard, we suggest that one of the potential causes might be that DF in eastern China has developed earlier and become more mature, and a portion of its incremental effect has been released ahead of time while the remaining progress space is smaller, which has reduced its influence on LIE. In contrast, the beneficial effect of digital banking on LIE is higher in the central and western area since its development there are nascent and weak, and their incremental effect are just beginning to emerge.

**TABLE 11:** Heterogeneity analysis.

	Region			Property rights	
	(1) East	(2) Central	(3) West	(4) State-owned	(5) private
	
	LIE	LIE	LIE	LIE	LIE
DF	–0.228** (–2.36)	–0.369*** (–4.65)	–0.366*** (–3.38)	–0.179* (–1.65)	–0.306*** (–6.99)
Constant	1.017*** (3.78)	0.538** (3.25)	0.975*** (5.91)	1.252*** (4.24)	0.676*** (9.33)
Control	Yes	Yes	Yes	Yes	Yes
Year	Yes	Yes	Yes	Yes	Yes
Industry	Yes	Yes	Yes	Yes	Yes
Region	Yes	Yes	Yes	Yes	Yes
Adj.R^2^	0.106	0.153	0.190	0.091	0.162
N	17039	3779	2685	8703	14800

***, **, and * Denote statistical significance at the 1%, 5%, and 10% levels, respectively. T-statistics are based on robust standard errors and are presented in parentheses.

Secondly, we divide the enterprises into state-owned and private enterprises based on the nature of property rights, and then run group regression. The outcomes are displayed in the columns (4)–(5) of Table 11, DF is significantly negatively correlated with LIE at the 1% level. These indicate that DF has a positive impact on the LIE of firms with different property rights, but its effect on the LIE of private enterprises is slightly larger than that of state-owned enterprises, which is consistent with the existing literature. To further explore the reason, in the Chinese institutional context, corporate financing behavior is often influenced by government intervention, and it has been easier for state-owned firms to obtain supportive credit subsidies (Shen et al., 2012), while private enterprises face stronger financing constraints (Jin et al., 2012), and thus private enterprises are more sensitive to labor costs. With the growth of DF, the financing constraints of private enterprises have been eased continuously. When there is a labor shortage, private firms might obtain more funds through financing to make up for the labor gap. In this process, LIE has also improved.

## Conclusion and policy recommendations

### Conclusion

As an emerging financial model, DF has far-reaching effects on corporate resource allocation. Alongside the rise of problems like an aging work force and a supply shortfall, we are concerned about whether DF will have an effect on firms’ LIE. Using the 2011–2020 data from Chinese A-share listed businesses to conduct our research, we derive the following main conclusions.

(1) The digital financial inclusion index, as well as its sub-dimensions -the breadth of coverage and depth of usage, has a stable and significant positive influence on corporate LIE. Consequently, the growth of DF improves the corporate LIE.
(2) DF improves the corporate LIE by affecting the financing constraints and human capital structure of firms. Specifically, DF effectively broadens an enterprises’ source of funds, improves financing efficiency, alleviates financing constraints and facilitate the financing of labor shortfalls, consequently improving corporate LIE. In the meantime, DF promotes firms’ R&D investment and technological innovation, resulting in a high demand for talents and driving enterprises to optimize their human capital structure. Then, DF promotes the digital transformation of enterprises, and intelligent operations replace simple and repetitive work, reducing personnel redundancy and optimizing the human capital structure. In addition, DF brings convenience for employees to improve their academic qualifications, and the increase in the number of on-the-job postgraduates also contributes to the optimization of the human capital structure of firms, improving LIE.
(3) The boosting influence of DF on LIE varies depending on the economic environment and the nature of firm. The western area with a poor economy is where this boosting impact performs best. This implies that the growth of DF may narrow the gap of LIE between regions, which provides a good opportunity for the balanced development of human capital in different regions. Additionally, the talent scarcity and financial constraints are the problems that private enterprises frequently encounter more acutely than those faced by state-owned enterprises. The growth of DF effectively alleviates these problems, so the boosting impact of DF is stronger in private enterprises than in state-owned ones.

### Policy recommendations

In light of the conclusions of the aforementioned research, we propose the following policy recommendations.

To address the crisis of insufficient labor resources and thus support the improvement of LIE, the government should strengthen the growth of DF. Specifically, first, the government could accelerate the growth of new kinds of infrastructure, including as big data, AI, and 5G, in addition to using the new generation of digital information technology to improve the conventional financial infrastructure. Second, the government should formulate logical policies to encourage the increase of DF, direct financial organizations to expand the breadth and depth of financial services, encourage them to create effective loan programs and financing options for businesses to address labor shortages. Enterprises should simultaneously seize the development opportunities offered by digital inclusive finance, actively complete digital transformation, apply digital technology to all facets of procurement, R&D, production, and sales, improve the level of enterprise information, obtain credit funds, lower financing costs, and create conducive conditions for enterprises to increase LIE. Third, traditional regulation is facing new challenges with the growth of DF, which is a fresh financial model. To eliminate financial risks and provide a welcoming environment for the growth of DF, the government should strengthen the regulatory framework. For instance, fraud in the process of digital financial inclusion might be found and harshly punished by the government using digital technology.

Raise the level of human capital and streamline its distribution. Specifically, first, the government should concentrate on developing creative technical talent, particularly in the digital sector. For example, cutting-edge disciplines like big data and artificial intelligence should be opened in colleges and universities to cultivate more excellent talents for digital development. Second, to promote the level of local human capital and contribute to local development, each region should create policies for introducing talent according to local conditions, attract more highly educated and skilled talents to settle down with good benefits and treatment. Third, companies should develop a scientific hiring and firing system to reduce unnecessary employee redundancy, recruit talented and skilled employees, and assign them to appropriate positions so as to enhance their LIE.

Last but not least, it should be emphasized that in the process of developing DF and optimizing human capital structure, the growth of enterprises in different regions and of different nature should be coordinated. The government should provide more policy support to economically depressed regions and private businesses in order to help them raise their levels of digitalization and human capital, which will increase the LIE.

### Limitations and future research

There are still some notable limitations of our paper. Firstly, DF, as a macro policy variable, has a complex influence path on corporate LIE. Due to a limitation of space, only two intrinsic paths, financing constraints and human capital structure, are covered in this paper. In order to further understand how DF influences corporate LIE, future study might examine more pathways of DF on the corporate LIE. Secondly, as digitalization deepens and technological advances with time, the concepts and specific initiatives related to DF are also expanding and updating. As a result, we can include more factors in upcoming studies, such as the digital economy and the digital governance. Thirdly, China is a socialist country with unique market economy policies. This indicates that its corporate governance and finance systems are different compared to those of other countries (Khan et al., 2021). The fact that our study is conducted in China may restrict how broadly the findings may be applied. Therefore, to increase the generalizability of the findings, further study might be carried out in the context of other developing and developed nations.

## Data availability statement

The raw data supporting the conclusions of this article will be made available by the authors, without undue reservation.

## Author contributions

JY: conceptualization, methodology, formal analysis, data curation, and writing – original draft. YJ: conceptualization, software, validation, and writing – review and editing. HC: validation, investigation, and writing – review and editing. SG: supervision. All authors contributed to the article and approved the submitted version.

## Footnotes

1 CSMAR database (China Stock Market & Accounting Research Database) is a research-oriented and accurate database in the field of economics and finance developed in combination with China’s actual national conditions, and is widely used in academic research in China.

2 The FC index in this paper is directly obtained from the CSMAR Database.

## Appendix

**TABLE A1:** The definition of variables.

Variables	Definition
LIE	Labor investment efficiency, See model (1) for details.
DF	The Digital Inclusive Financial Index of city-level published by the Digital Finance Research Center of Peking University.
Breadth	The breadth of the Digital Inclusive Financial Index of city-level.
Depth	The depth of the Digital Inclusive Financial Index of city-level.
FC	Index of external finance constraints of a firm estimated in one year. See Fee et al. (2009) for details.
HT	The proportion of employees with master’s degree or above in the total number of employees in the firm.
GDP	Natural logarithm of GDP.
ILI	The industry Lerner index.
Age	Current year minus year of establishment.
Assets	Natural logarithm of total assets.
Lev	Total liabilities divided by total assets.
FA	Natural logarithm of net fixed assets.
Growth	Annual growth rate of operating revenue.
CR	Quick assets divided by current liabilities.
LI	Number of employees at the beginning of the year divided by total assets.
Capital	Total assets divided by revenue from main business.
H10	The shareholding ratio of the top ten shareholders.
DUAL	A dummy variable that equals one if a firm’s chairman and CEO are the same person and zero otherwise.

**TABLE A2:** Bootstrap test.

Independent variable	Mediating variables	Dependent variables	Effect	Estimated effect	95% CI	
					LLCI	ULCI
DF	FC	LIE	Indirect	–0.046*** (–4.83)	–0.019	–0.008
			Direct	–0.392*** (–11.87)	–0.470	–0.338
DF	HT	LIE	Indirect	–0.071** (–2.01)	–0.022	–0.000
			Direct	–0.443*** (–13.97)	–0.505	–0.381

***, **, and * Denote statistical significance at the 1, 5, and 10% levels, respectively. T-statistics are based on robust standard errors and are presented in parentheses. 95% CI refers to the bias-corrected bootstrapped confidence interval.
